# Oxidative Stress-Involved Mitophagy of Retinal Pigment Epithelium and Retinal Degenerative Diseases

**DOI:** 10.1007/s10571-023-01383-z

**Published:** 2023-07-01

**Authors:** Si-Ming Zhang, Bin Fan, Yu- Lin Li, Zhao-Yang Zuo, Guang-Yu Li

**Affiliations:** Department of Ophthalmology, Second Norman Bethune Hospital of Jilin University, Changchun, 130000 China

**Keywords:** Retinal pigment epithelium, Oxidative stress, Mitophagy, Age-related macular degeneration, Diabetic retinopathy

## Abstract

The retinal pigment epithelium (RPE) is a highly specialized and polarized epithelial cell layer that plays an important role in sustaining the structural and functional integrity of photoreceptors. However, the death of RPE is a common pathological feature in various retinal diseases, especially in age-related macular degeneration (AMD) and diabetic retinopathy (DR). Mitophagy, as a programmed self-degradation of dysfunctional mitochondria, is crucial for maintaining cellular homeostasis and cell survival under stress. RPE contains a high density of mitochondria necessary for it to meet energy demands, so severe stimuli can cause mitochondrial dysfunction and the excess generation of intracellular reactive oxygen species (ROS), which can further trigger oxidative stress-involved mitophagy. In this review, we summarize the classical pathways of oxidative stress-involved mitophagy in RPE and investigate its role in the progression of retinal diseases, aiming to provide a new therapeutic strategy for treating retinal degenerative diseases.

**Graphical Abstract:**

The role of mitophagy in AMD and DR. In AMD, excessive ROS production promotes mitophagy in the RPE by activating the Nrf2/p62 pathway, while in DR, ROS may suppress mitophagy by the FOXO3-PINK1/parkin signaling pathway or the TXNIP-mitochondria-lysosome-mediated mitophagy.

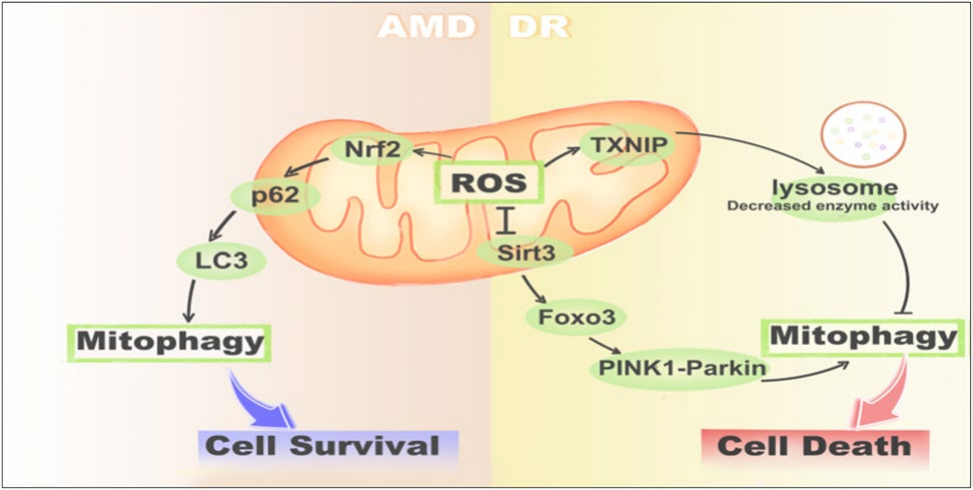

## Introduction

The retinal-pigmented epithelium (RPE) is located in the outer layer of the retina between the photoreceptors and the choroid, which constitutes the blood-retina outer barrier (Simó et al. 2010). The RPE, as a fundamental component of the retina, plays a vital role in visual function, such as selectively transporting metabolites and nutrients between the retina, and blood circulation through the Bruch's membrane, maintaining the structure and function of photoreceptor cells, phagocytizing the shed outer segments of photoreceptors (POS), and participating in the conversion of all-trans-retinal and 11-cis-retinal in the visual cycle (Naso et al. 2020; Coffe et al. 2006; Khristov et al. 2018). Therefore, the RPE contains large numbers of mitochondria to meet high metabolic demands, which also means to consume more oxygen and produce massive reactive oxygen species (ROS) (Mazzoni et al. 2014).

The dysfunction of the RPE is associated with various retinal diseases, especially in the progression of age-related macular degeneration (AMD) and diabetic retinopathy (DR). The high oxygen consumption, the phagocytosis of lipid peroxidation products from the POS, and prolonged light irradiation are the major risk factors that induce oxidative-stressed injury in the RPE. Mitochondria, as the energy factories that produce ATP, also generate a large amount of intracellular ROS as a byproduct of oxidative phosphorylation (Datta et al. 2017). Under physiological conditions, ROS and reactive nitrogen species (RNS) may function as second messengers in signal transduction and play critical roles in maintaining normal cellular function. However, the excessive ROS and RNS may also result in the imbalance of the intracellular redox system, which further leads to the peroxidation of cellular components, such as lipids, proteins, and deoxyribonucleic acid (DNA), as well as cause the dysfunction of the mitochondria (Guo et al. 2013). Under normal physiological conditions, the dysfunctional mitochondria can be selectively removed via oxidative stress-involved mitophagy to maintain cellular homeostasis and promote survival of cell. However, under stress conditions, such as in AMD or DR, the process of mitophagy in RPE is impaired, resulting in the excessive accumulation of dysfunctional mitochondria, which further leads to the release of apoptosis-related factors such as cytochrome C and triggers a mitochondrial-dependent death cascade (Montava-Garriga and Ganley 2020; Skeie et al. 2021). Thus, regulating oxidative stress-involved mitophagy is an essential strategy for protecting RPE against various pathological damages, which also may play a crucial role in preventing the progression of retinal degenerative diseases.

In this review, we summarize the classical signaling pathways of oxidative stress-involved mitophagy in RPE and illustrate the role of mitophagy in the progression of AMD and DR, aiming to develop a new therapeutic strategy for treating retinal degenerative diseases.

## Autophagy and Mitophagy

As an evolutionarily conserved process, autophagy removes damaged intracellular components, such as proteins, nucleic acids, and organelles. In the process of autophagy, the dysfunctional proteins and organelles are transported into lysosomes, where the macromolecules, such as proteins, nucleic acids, carbohydrates, and lipids, are degraded and recycled (Bento et al. 2016). There are three major classes of autophagy: (1) macroautophagy, (2) microautophagy, and (3) chaperone-mediated autophagy (Boya et al. 2013; Kaushik and Cuervo 2012; Sahu et al. 2011). As a multistep process, the initiation of autophagy first requires that autophagy-related proteins (ATGs) are recruited to the phagophore assembly site (PAS) to nucleate the isolation membrane of a cup-shaped phagophore. Next, the elongation of the isolation membrane eventually seals into a double-membraned autophagosome, in which the autophagic cargo is engulfed. The autophagosome then travels along microtubules to the lysosome, where the two fuse to form an autolysosome. Finally, the autophagic cargo is released within the lysosome, and it is hydrolyzed and recycled. Although autophagy was regarded as a non-selective degradation pathway, currently it is widely accepted that autophagy is a unique process for selectively removing unnecessary intracellular proteins, organelles, and pathogens (Abada and Elazar 2014; Dikic and Elazar 2018).

As an important type of autophagy, mitophagy is strictly regulated by multiple signaling pathways that play a crucial role in maintaining mitochondrial and cellular homeostasis under various stress conditions. The discovery that damaged mitochondria are selectively removed by autophagosomes after losing membrane potential suggested that mitophagy may be responsible for this phenomenon (Lemasters et al. 1998). The intracellular senescent or damaged mitochondria can be selectively removed and degraded via mitophagy. A basic procedure necessary for preserving mitochondrial fitness in a variety of cell types is mitochondria-specific autophagy, or mitophagy. In particular, mitophagy improves mitochondrial quality control by removing malfunctioning mitochondria in a targeted manner. However, if the process of mitophagy is impaired, the accumulation of dysfunctional mitochondria may result in the release of apoptotic factors, such as cytochrome C, apoptosis-inducing factor (AIF), and endo G, which may trigger a mitochondria-dependent programmed cell death (Bayir and Kagan 2008). Studies reported that a deficiency in mitophagy is closely related to the pathogenesis of neurodegeneration, metabolic diseases, and ischemia–reperfusion injury. Despite the fact that mitophagy was once thought to be a quality control mechanism to assess mitochondrial damage, we now know that mitophagy also plays a crucial role in various physiological processes, such as the fertilization of egg cells, the differentiation of retinal ganglion cells, and the maturation of reticulocytes (Saito and Sadoshima 2015; Gkikas et al. 2018; Sandoval et al. 2008).

## Signaling Pathways of Mitophagy

**Fig. 1:**
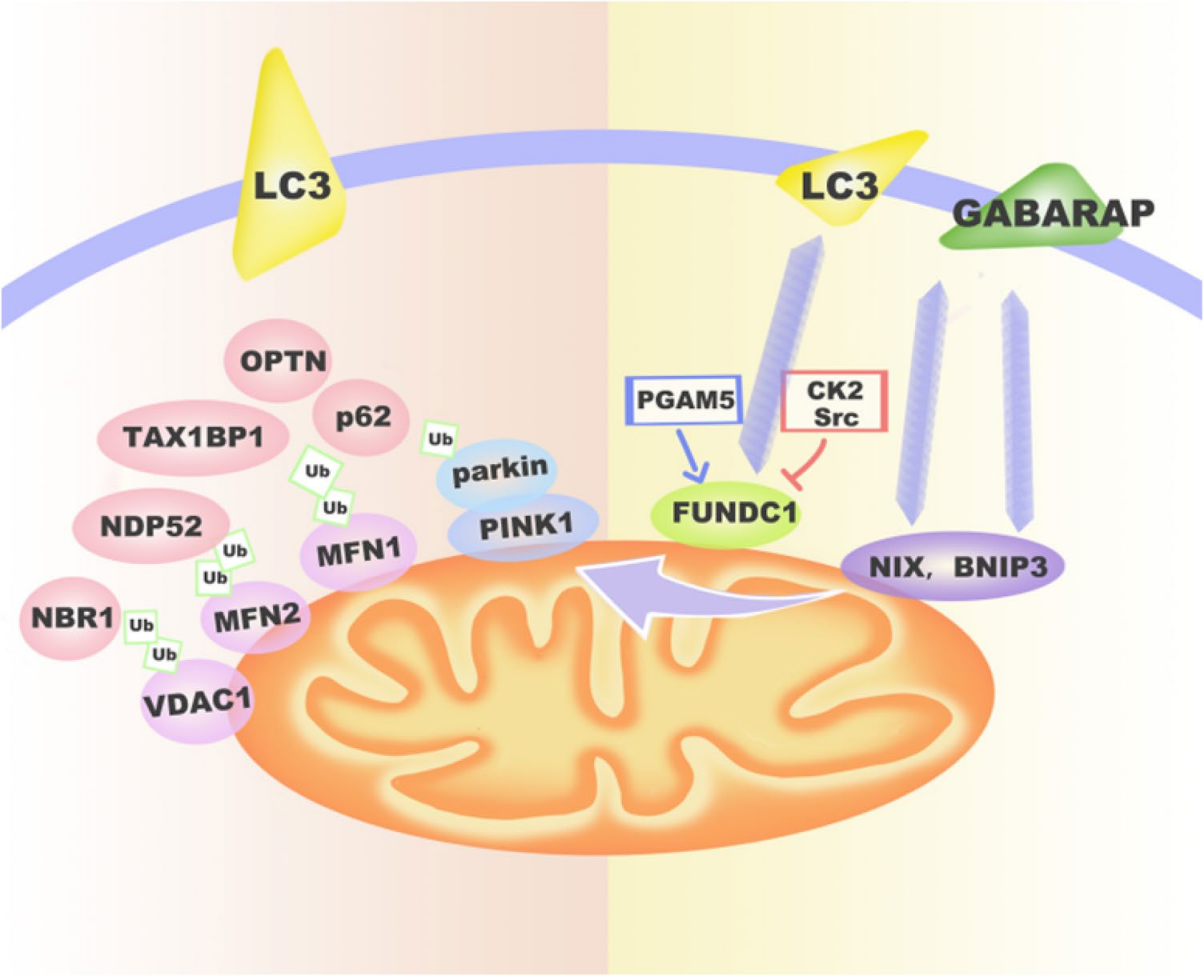
The signaling pathways of mitophagy. (1) Non-Receptor-Mediated Mitophagy: Activation of PINK1 leads to recruitment of ubiquitin and Parkin. Parkin ubiquitinates and phosphorylates mitochondrial proteins (such as VDAC1, MFN1, and MFN2), and this initiates receptor adaptor protein recruitment (p62, NDP52, OPTN, TAX1BP1, and NBR1). These adaptor proteins interact with LC3 to form the autophagosome. (2) Receptor-Mediated Mitophagy: Mitochondrial receptor proteins (BNIP3, NIX, FUNC1) are ubiquitinated and phosphorylated. This facilitates their interaction with LC3 and GABARAP for autophagosome formation

There are two major processes in mammalian cells through which mitophagy is conducted; one is ubiquitin dependent and the other is receptor dependent (Fig. 1).

### PINK1/Parkin-Mediated Ubiquitin-Dependent Mitophagy

Bulleted lists look like this: The well-studied ubiquitin-dependent mitophagy process occurs via the phosphatase and tensin homolog (PTEN)-induced kinase 1 (PINK1)/parkin pathway, which is predominantly mediated by the mitochondrial-targeted serine/threonine kinase PINK1 and the cytosolic ubiquitin E3 ligase (Parkin). In healthy mitochondria, cytosolic PINK1 encoding a mitochondrial-targeting signal (MTS) and a single membrane-spanning domain, is transported to the inner mitochondrial membrane by the translocase of outer mitochondrial membrane (TOM) complex and translocase of inner mitochondrial membrane (TIM) complex, where it is cleaved and degraded by proteases, such as the inner membrane presenilin-related rhomboid-like protease (PARL) (Matsuda et al. 2010). However, PINK1 can act as a molecular sensor for damaged mitochondria. When mitochondria are depolarized, PINK1 rapidly accumulates and spans the outer mitochondrial membrane (OMM) with its kinase domain facing the cytosol (Zhou et al. 2008). When PINK1 is stabilized on the OMM, it further recruits Parkin to the impaired mitochondria and phosphorylates it at Ser65 (Okatsu et al. 2012; Ordureau et al. 2014; Sauve et al. 2015; Matsuda et al. 2010). The activated Parkin combines ubiquitin (Ub) chains with the OMM using multiple-ubiquitinating mitochondrial proteins, such as mitofusin-1 (MFN1), mitofusin-2 (MFN2), and voltage-dependent anion channel-1 (VDAC1) (Okatsu et al. 2012; Novak 2012). The ubiquitinated structure is recognized by autophagic receptors, including the sequestosome-like protein receptors (receptors optineurin (OPTN), Neighbor of Brca1 (NBR1), nuclear dot protein 52 (NDP52), Tax1-binding protein 1 (TAX1BP1), and Sequestosome1/p62 (SQSTM1/p62)) (Padman et al. 2019; Lazarou et al. 2015; Heo et al. 2015), promoting the transportation of the damaged mitochondria into autophagosomal vesicles. In addition, PINK1 also mediates the phosphorylation of Ub, which recruits the autophagic receptors NDP52 and optineurin to trigger Parkin-independent mitophagy (Padman et al. 2019).

PINK1/Parkin-mediated mitophagy plays a crucial role in mitochondrial homeostasis and is involved in mitochondrial dynamics, biogenesis, transport, and the recruitment of autophagic machinery.

### BNIP3 and NIX-Mediated Receptor-Dependent Mitophagy

BCL2-interacting protein 3 (BNIP3) and its homologous proteins BNIP3-like (BNIP3L)/NIX are BH3-only proteins located in OMM and belong to the BCL2 family. BNIP3 directly interacts with the autophagy-related protein LC3 by an N-terminal LC3-interacting region (LIR) to mediate ubiquitination-independent mitophagy (Quinsay et al. 2010b, 2010a). The phosphorylation of Ser17 and Ser24 near the LIR motif of BNIP3 enhances the interaction with LC3. Under stress conditions, BNIP3 triggers receptor-dependent mitophagy on the OMM through a homodimer formed by its C-terminal transmembrane (TM) domain. BNIP3 may regulate mitochondrial dynamics by promoting the fission of damaged mitochondria, which is also a necessary step for mitophagy. Under hypoxic conditions, BNIP3 is activated by hypoxia-inducible factor (HIF) or forkhead homeobox type O (FOXO), leading to hypoxia-induced mitophagy (Hanna et al. 2012). NIX also contains a LIR motif that interacts with LC3A, LC3B, and GABA Type A Receptor-Associated Protein (GABARAP). The phosphorylation of NIX at Ser34 and Ser35 near the LIR motif enhances its interaction with LC3 (Jung et al. 2019). In the process of mitochondrial oxidative phosphorylation (OXPHOS), NIX combines with Rheb small GTPase to promote mitophagy. In addition, previous studies reported that both NIX and BNIP3 may regulate PINK1/Parkin-mediated mitophagy to sustain mitochondrial homeostasis by influencing the recruitment of Parkin, which indicates that there is a crosstalk between the receptor-dependent pathway and the PINK1/Parkin-mediated ubiquitin pathway (Palikaras et al. 2018; Ding et al. 2010).

### FUNDC1-Mediated Receptor-Dependent Mitophagy

FUN14 domain containing 1 (FUNDC1), as an OMM protein, interacts with LC3 through its LIR motif to activate mitophagy. Under non-stressed conditions, the activity of FUNDC1 is suppressed by Src kinase-induced phosphorylation at Tyr18, while cells under stress conditions, the Src kinase is inactivated, which leads to dephosphorylation of FUNDC1 and further promotes mitophagy (Liu et al. 2012). In addition, under hypoxic conditions, Phosphoglycerate mutase family member 5 (PGAM5) disrupts the interaction between FUNDC1 and Optic atrophy 1 (OPA1) via dephosphorylating the former at Ser13 and inhibits mitochondrial fusion, thereby promoting FUNDC1-Dynamin-related protein 1 (DRP1) interaction and mitochondrial fragmentation (Chen et al. 2016; Wu et al. 2016). FUNDC1 may also interact with unc-51-like kinase 1 (ULK1) and promote its relocation to mitochondria, inducing mitophagy (Wu et al. 2014).

## Oxidative Stress Damage in RPE

As one of the tissues that consumes the most oxygen, the retina is responsible for receiving light signals to form vision, yet prolonged and excessive exposure to light irradiation may also trigger photo-oxidative reactions, resulting in increased production of ROS (Beatty et al. 2000). These ROS include superoxide radical (O^2·−^), hydrogen peroxide, hydroxyl radical (OH·), and singlet oxygen (1O^2^). In normal cells, ROS may function as an intracellular second messenger and play a crucial role in maintaining physiological activities (Finkel 2011; Fanjul-Moles and López-Riquelme 2016). When cells are exposed to exogenous oxidative stressors, including ultraviolet light, ionizing radiation, or cigarette smoke, excessive ROS are generated in cells, which lead to the imbalance of the redox system, resulting in oxidative damage to cellular lipids, proteins, and nucleic acids.

**Fig. 2:**
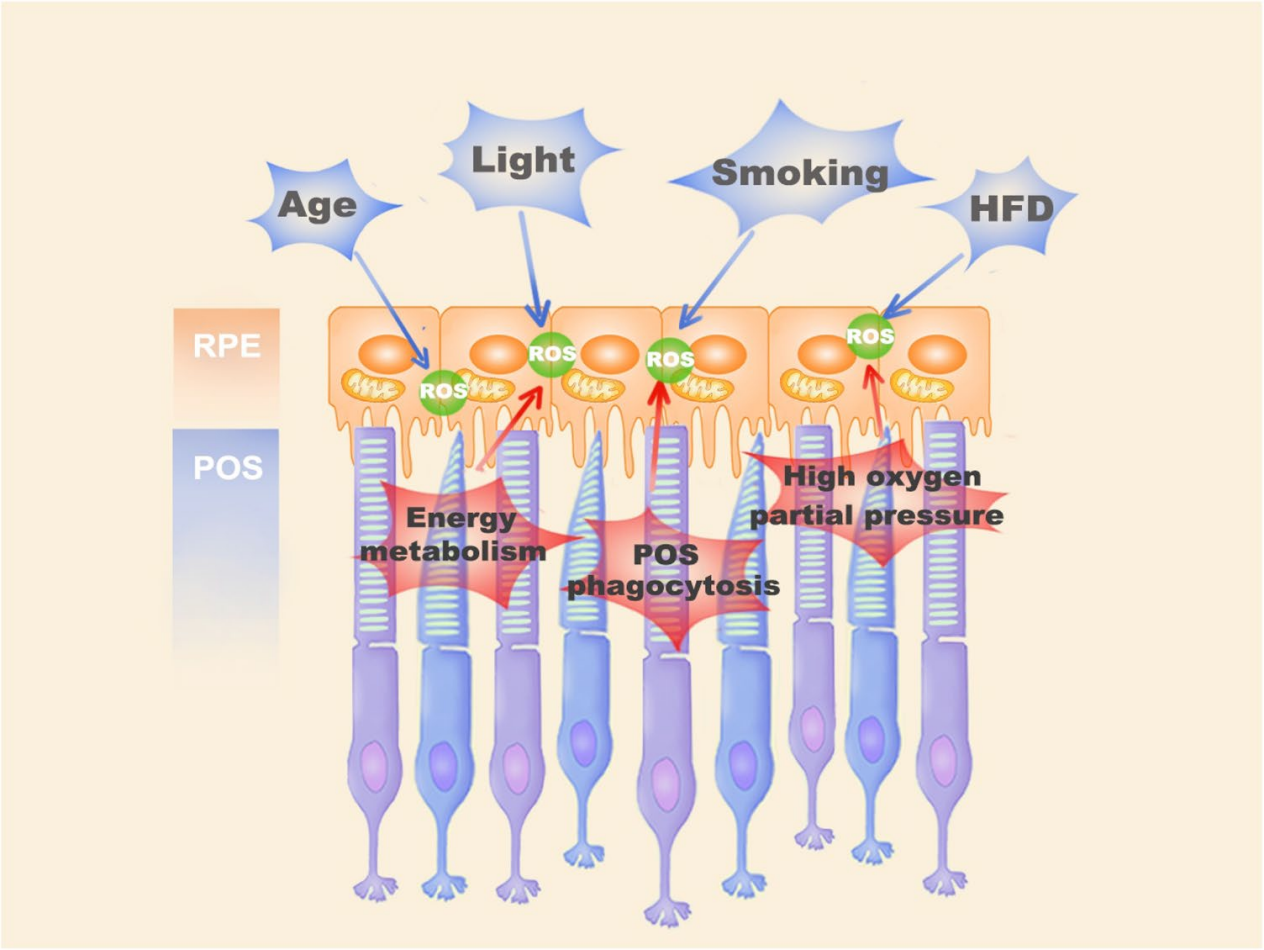
The role of oxidative stress in RPE. Aging, light injury, cigarette, and HFD are the risk factors. Overactive energy metabolism and excessive signal transduction in RPE and photoreceptor cells produce many ROS. Daily phagocytosis of POSs in RPE cells is also an important source of ROS. In addition, RPE is continuously exposed to high oxygen pressure

The RPE is a monolayer of cells located on Bruch's membrane between the neurosensory retina and choroid, where it plays an important role in maintaining the functions of the retina (Marmorstein 2001; Miller and Steinberg 1977; Rizzolo 1997; Strauss 2005). Due to its high energy metabolism and energy demand, the RPE is rich in mitochondria, which allow it to produce sufficient Adenosine 5ʹ-triphosphate (ATP) (Jager et al. 2008) (Fig. 2). In addition, the RPE needs to exchange nutrients and waste with the blood in the choroid, which continuously exposes the RPE to an environment with high oxygen pressure (70–90 mmHg) (Winkler et al. 1999). Furthermore, another important function of the RPE is to phagocytose the POS shed from photoreceptors that contain photosensitive groups, various oxidants, and unsaturated fatty acids, which leads to the increased production of ROS in the RPE (Ershov and Bazan 2000). In addition, aging is another risk factor that induces oxidative-stressed damage in the RPE. With aging, the number of dysfunctional mitochondria increases in the RPE, leading to a significant increase in ROS generation (Jensen 1966). Jarrett et al*.* found that age-related lipofuscin (a photo-oxidative substance in RPE), 8-oxoguanine (the main product of oxidative DNA damage), mitochondria DNA (mtDNA) damage, carboxyethylpyrrole (CEP, oxidative fragments of docosahexaenoic acid), as well as 4-hydroxynonenal (4-HNE) and malondialdehyde (MDA, a product of lipid peroxidation) were significantly increased in the aging retina (Jarrett and Boulton 2012; Rahman and MacNee 1996). Smoking also plays a role in promoting oxidative stress in the RPE (Cross et al. 1993). Lykkesfeldt et al*.* found that cigarette smoke contains various intensive oxidants, which may cause the depletion of the ascorbic acid and sulfhydryl groups of proteins in RPE, leading to an imbalance in the antioxidant system and oxidative damage to DNA, lipids, and proteins. Moreover, a high-fat diet also has been demonstrated as a risk factor to induce oxidative stress in the RPE (Kaarniranta et al. 2019). Ebrahimi et al. found that feeding a long-term high-fat diet to mice resulted in the suppression of Wingless (Wnt) signaling in the RPE, leading to damage to the Nuclear factor (erythroid-derived 2)-related factor 2 (Nrf2)-dominated antioxidant network (Ebrahimi et al. 2018).

## Signaling Pathway of Oxidative Stress-Involved Mitophagy in RPE

Oxidative stress damage may lead to the dysfunction of mitochondria, and the accumulation of damaged mitochondria may further trigger mitophagy. In addition, intracellular ROS also participates in the regulation of mitophagy through multiple signaling pathways.

### PINK1/Parkin-Mediated Ubiquitin-Dependent Mitophagy

Sirt3 is the most important NAD^+^-dependent sirtuin in the mitochondria. Through deacetylation, Sirt3 regulates mitochondrial energy metabolism and ROS production and suppresses cellular oxidative damage (Rangarajan et al. 2015). FOXO3a is a transcription factor that increases the expression of anti-oxidative genes and decreases intracellular ROS levels, protecting cells against oxidative stress-induced death apoptosis (Checler et al. 2017). Jacobs et al. showed that overexpression of Sirt3 increases deacetylated FOXO3a, resulting in reduced phosphorylation, ubiquitination, and degradation, which is involved in regulating oxidative stress and autophagy of cells (Jacobs et al. 2008). Mei et al*.* found that PINK1 may function as an essential downstream mediator of FOXO3a, and the activation of it may result in a significant upregulated expression of PINK1 and parkin, which induce increased mitophagy (Mei et al. 2009). Hu et al. found that decreased Sirt3 and suppressed mitophagy were detected in the corneal epithelium of C57BL/6J-Ins2Akita (Ins2^Akita/+^) (a diabetic animal model), while overexpression of Sirt3 induced the activation of Foxo3a, and further resulted in the activation of mitophagy via the PINK1-Parkin pathway (Hu et al. 2019). Li Huang et al*.* found that under high glucose (HG) conditions, the expression of Sirt3 in the RPE is decreased, which in turn induced an increased level of intracellular ROS, but the FOXO3a/PINK1–Parkin-mediated mitophagy pathway was attenuated. In addition, Li Huang et al*.* found that the overexpression of Sirt3 triggered mitophagy via the FOXO3a/PINK1–Parkin pathway to suppress apoptosis, which protected the RPE under HG conditions, yet Sirt3-induced protection was blocked by the genetic silencing of PINK1 (Huang et al. 2022). Thus, these results suggest that Sirt3 may play an anti-oxidative role in regulating the production of ROS, and it may trigger mitophagy via the FOXO3a/PINK1–Parkin pathway to maintain the homeostasis of intracellular mitochondria and protect the RPE against oxidative stress injury.

### TXNIP-Mitochondria-Lysosome-Mediated Mitophagy

Thioredoxin-interacting protein (TXNIP) is a kind of thioredoxin (TRX)-binding protein that plays a role in mediating oxidative stress, which results in damage and apoptosis. Previous studies have shown that the increased expression of TXNIP in mitochondria (including pancreatic beta, kidney, and retinal cells) is detected under HG conditions, which causes damage and dysfunction to mitochondria (Singh 2013; Cheng et al. 2006; Perrone et al. 2009). Su et al*.* reported that HG significantly upregulated the expression of TXNIP and increased the level of ROS, but downregulated the expression of PINK1 and Parkin and suppressed mitophagy, all of which contributed to the death of rat pheochromocytoma (PC12) cells. They also reported that siRNA-mediated knockdown of TXNIP significantly reduced intracellular ROS, activated mitophagy, and attenuated the damage of PC12 cells under HG conditions (Su et al. 2020). These results indicated that PINK1/Parkin-mediated mitophagy could be activated by inhibiting TXNIP under HG conditions, which exerted a protective role in PC12 cells. In addition, Huang et al*.* reported that HG caused the dysfunction of mitochondria and suppressed mitophagy, while inhibiting TXNIP mitigated dysfunction and promoted mitophagy in HK2 cells in a rat diabetic nephropathy model (Huang et al. 2014). However, Devi et al*.* showed that silencing the expression of TXNIP with shRNA inhibited hydrogen peroxide-induced mitophagy in APRE-19 cells and exerted a protective effect, but that HG led to increased expression of TXNIP and mitophagy flux in cultured ARPE-19 cells. However, when the mitophagy flux exceeded the capacity of lysosomes, their enzyme activity was suppressed, which caused the inhibition of mitophagy (Devi et al. 2019). Therefore, silencing the expression of TXNIP with shRNA significantly inhibited mitophagy under hyperglycemic conditions in APRE-19 cells and maintained mitochondrial homeostasis and function. Similarly, Singh et al*.* showed that HG induced an increased level of ROS and upregulated expression of TXNIP in rat Müller cells, but inhibition of TXNIP with siRNA exerted a protective effect (Singh 2013). These results suggest that regulation of the mitochondria–lysosome–mitophagy axis through TXNIP modulation may play a protective role in oxidative stress-induced RPE damage.

### Nrf2-P62-Mediated Mitophagy

The transcription factor Nrf2 (nuclear factor-related factor 2), a member of the basic leucine zipper family, is involved in mitochondrial biogenesis, regulates intracellular ROS levels, and plays a key role in maintaining cellular homeostasis (Bellezza et al. 2010). Under normal physiological conditions, Nrf2 is degraded by binding to the E3 ubiquitin ligase Kelch Like ECH Associated Protein 1 (KEAP1), while under oxidative stress conditions, it binds to the promoter of p62 and stimulates its expression (Bellezza et al. 2018). The p62 gene encodes an LIR motif that interacts with LC3 to trigger mitophagy. Therefore, Nrf2 promotes mitophagy by regulating p62 under oxidative stress conditions (Wang et al. 2016a; Myeku and Figueiredo-Pereira 2011). Zhao et al*.* found that the knockout of Nrf2 resulted in swollen mitochondria in the RPE of mice, which was accompanied by increased autophagy-related vacuoles, and damaged mitochondria were often found adjacent to autophagy vacuoles. In addition, the mice with Nrf2 knockout exhibited typical features of retinal degeneration, such as accumulated drusen and lipofuscin, as well as increased inflammatory proteins (Zhao et al. 2011). These results suggest that Nrf2 knockout impairs mitophagy in RPE cells, which promotes retinal degeneration. Felszeghy et al*.* found that the PGC-1α and Nrf2 double-knockout mouse model had increased expression of mitophagy-related proteins, and also that lysosome accumulation was present in RPE cells. However, mitophagy flux failed to increase, indicating that the mitophagy pathway was impaired because the downstream signals of mitophagy were inhibited. Furthermore, the double knockout promoted RPE degeneration in mice and resulted in visual dysfunction similar to that seen in AMD (Felszeghy et al. 2019).

## RPE Mitophagy and Retinal Diseases

### Age-Related Macular-Mediated Degeneration

AMD is the leading cause of blindness among older adults in developed countries, with more than 11 million people living with it in the United States (Pennington and DeAngelis 2016). Various environmental and genetic factors play crucial roles in the pathogenesis and progression of AMD, including aging, light exposure, genetics, and poor lifestyle (Black and Clark 2016; Blasiak et al. 2019). Damage of oxidative stress to the RPE is a key pathological feature in AMD (Cai et al. 2000; Cai and McGinnis 2012; Jarrett and Boulton 2012). Accumulating evidence indicates that the increased production of ROS, the excessive accumulation of nonfunctional mitochondria, and oxidative stress-triggered mitophagy in the RPE are key in the progression of AMD (Kaarniranta et al. 2020).

When analyzing mitochondrial genomes, Terluk et al*.* found that the DNA damage of mitochondria in the RPE in AMD patients was increased, which was correlated with the severity of AMD (Terluk et al. 2015). In addition, damage to the structure of the inner and outer mitochondrial membranes, abnormally sized mitochondria, and a reduced number of mitochondria were also detected in the RPE of AMD patients (Bianchi et al. 2013; Brown et al. 2018). Gurubaran et al. reported that a double knockout of NFE2L2/PGC-1α^−/−^ resulted in an increased level of oxidative stress-related markers, the accumulation of damaged mitochondria, and the accumulation of lysosomal lipofuscin in the RPE in the mice, which is consistent with findings found in AMD patients (Gurubaran et al. 2020; Felszeghy et al. 2019; Kaarniranta et al. 2020). In addition, the double-knockout mice also had upregulated mitophagy-related proteins, LC3B, PINK1, and Parkin, as well as accumulated dysfunctional mitochondria (Gurubaran et al. 2020), suggesting that impaired mitophagy plays a role in the progression of dry AMD. In addition, Chang et al*.* reported that the Urban particulate matter (UPM) of air pollution particles is closely related to the progression of AMD (Chang et al. 2019). The air pollution particle UPM may cause an increased level of intracellular ROS and promote the expression of mitophagy-related proteins, PINK1, Parkin, and LC3I/II in RPE, and ROS-mediated mitophagy may play a role in UPM-induced retinal diseases (Lee et al. 2021). Stenirri et al*.* found that mutations of mitochondrial ferritin (FtMt) led to the dysfunction of FtMt in AMD patients (Stenirri et al. 2012), and its overexpression caused a decrease in mitochondrial membrane potential (MMP) and a reduction in OPA1, but promoted the fission of mitochondria (Wang et al. 2016b), suggesting that the overexpression of FtMt stimulated mitophagy to exert a protective effect in the RPE.

As an oxidative toxicant, NaIO3 selectively induces damage to the RPE, so it is used to establish in vitro AMD models (Wang et al. 2014; Balmer et al. 2015). Likewise, Bafilomycin A1 is an inhibitor of autophagy that blocks the fusion of autophagosomes and lysosomes at a late stage in autophagy, is often used to determine autophagic flux. Chan et al*.* found that compared with the treatment of Bafilomycin A1 alone, treatment with both Bafilomycin A1 and NaIO3 induced significant elevated ROS levels in ARPE-19, and was followed with a significant increase in LC3II and TOM20 co-labeling (Chan et al. 2019), suggesting that increased ROS could promote mitophagy in RPE. However, treatment of NaIO3-damaged RPE with the antioxidants NAC or Trolox leads to the level of cellular ROS being markedly reduced, mitochondrial fragmentation being significantly increased, and mitophagy being impaired, all leading to the death of ARPE-19 cells. Therefore, these results suggest that damaged mitochondria can be removed via ROS-mediated mitophagy in ARPE-19 cells and can mitigate cell death, thus, exhibiting a protective role in AMD.

### Diabetic Retinopathy

Microvascular lesions of the retina are a typical pathological feature of DR, and they manifest as the shedding of vascular endothelial cells and pericytes, the thickening of endothelial cell basement membrane, the leakage of blood, and the deposition of extravascular lipid and protein (Antonetti et al. 2006; Villarroel et al. 2009). However, previous studies have shown that long-term hyperglycemia leads to significant damage on the function and ultrastructure of retinal pigment epithelial cells, so injury to the RPE is an early pathological change in the progression of DR (Aizu et al. 2002; Bensaoula and Ottlecz 2001; Grimes and Laties 1980; Samuels et al. 2015). Bayir et al*.* found that there is significant loss and degeneration in RPE in the retina of diabetes rats, accompanied by morphological changes to organelles, such as nuclear shrinkage or the formation of an oval nucleus, an expanded or reduced endoplasmic reticulum, membrane folding, or changes in the distribution of melanosomes (Tarchick et al. 2016; Blair et al. 1984; Tso et al. 1980). In addition, HG downregulated the activities of glucose transporter-1 (GLUT-1) and Na^+^/K^+^-ATPase in RPE, damaging retinal glucose transport capacity and metabolism (Kim et al. 2007; Bensaoula and Ottlecz 2001). In DR and cultured RPE with high glucose, the expression of interstitial retinol-binding protein (IRBP) and pigment epithelium-derived factor (PEDF) is downregulated (Garcia-Ramírez et al. 2009; Yang et al. 2012). Moreover, the retinal capillary leakage of DR may directly destroy the tight connection between RPE cells, resulting in the disorder of the ionic environment in the subretinal cavity, which severely affects the function of photoreceptor cells (Tonade and Kern 2021). In addition, Kanwar et al*.* found that the levels of glutathione, superoxide dismutase (SOD), and other antioxidant active molecules in the retinal mitochondria of diabetic mice were significantly reduced (Kanwar et al. 2007), which led to a compromised antioxidant defense capacity of RPE and to oxidative stress injury in the retina.

Mitochondrial dysfunction and oxidative-stressed injury play key roles in the early stage of DR (Masser et al. 2017). Kowluru et al. showed that there is significant damage to mtDNA, marked up-regulation of ROS, and dysfunctional mtDNA repair in diabetic retinopathy (Kowluru and Mishra 2015). Zhong et al*.* found that there is manifest vacuolated and disrupted lamellar cristae in the mitochondria in the retinal endothelial cells and neurons of DR at the ultrastructural level (Zhong and Kowluru 2011). Van Houten et al*.* also observed the increased mitochondrial fragmentation and decreased oxygen consumption rates in RPE cultured in HG condition (Van Houten et al. 2016). Thus, these results indicate that the accumulation of damaged mitochondria disrupts the homeostasis of RPE, leading to increased oxidative stress, a lack of ATP, and cell death. Zhang et al*.* reported that RPE cultured in HG (50 mM) had increased ROS and that it inhibited the expression of PINK1 and Parkin, which led to the suppression of mitophagy and cell death. Treatment with ROS scavengers or the overexpression of PINK1/Parkin attenuated the HG-induced damage in RPE, while treatment with cyclosporin A (CsA) (the inhibitor of mitophagy) exacerbated it (Zhang et al. 2019). These results demonstrate that there is a close correlation between elevated ROS, impaired mitophagy, and increased cell death in RPE under HG conditions, suggesting that scavenging ROS and restoring PINK1/Parkin-mediated mitophagy may be therapeutic in DR. Kuo et al*.* found that a mutation of mitochondrial DNA resulted in the elevation of ROS level, impaired mitophagy, and increased apoptosis in diabetes-prone hybrid cells, while treatment with the antioxidant NAC markedly ameliorated these changes (Kuo et al. 2016), suggesting that the elevated level of ROS contributes to dysfunctional mitophagy. Therefore, mtDNA variants in diabetes-prone cells may increase the oxidative damage to mitochondria, which in turn leads to mitochondrial dysfunction and impaired mitophagy.

NotoginsenosideR1 (NGR1) has anti-inflammatory properties and functions as a scavenger of ROS, and is used in the treatment of diabetic encephalopathy and diabetic microvascular disease (Lian et al. 2015; Zhang et al. 2018). Zhou et al*.* found that NGR1 treatment significantly reduced ROS generation and apoptosis in HG-induced retinal Müller-1 cells (rMC-1) and increased the expression of PINK1 and Parkin and the ratio of LC3II/LC3I, but downregulated the expression of SQSTM1/p62. In addition, NGR1 treatment increased the co-localization ratio of GFP-LC3 puncta and mitotracker in rMC-1 cells, indicating increased mitophagy. However, the knockdown of PINK1 with siRNA suppressed the NGR1-induced protection in rMC-1 cells under HG conditions. In addition, elevated ROS levels and a decrease in the ratio of LC3II/LC3I were also observed (Zhou et al. 2019), indicating that NGR1 protected rMC-1 cells through PINK1-mediated mitophagy under HG conditions. Therefore, these studies suggest that PINK1/parkin-mediated mitophagy may attenuate the oxidative damage and play an important role in the treatment of DR.

## Conclusion and Future Directions

Mitochondria are the energy synthesis factories of cells, and ROS are a byproduct of energy generation. As a second messenger, ROS plays an important role in cell signal transduction. However, with redox imbalance, excessive ROS leads to organelle damage, including mitochondria, endoplasmic reticulum, and the nucleus, which can trigger apoptosis. Through mitophagy, cells remove damaged mitochondria, reduce the production of intracellular ROS, maintain intracellular homeostasis, and promote cell survival. However, under different stress stimuli, ROS also regulates mitophagy through various signal pathways. For example, in AMD, excessive ROS production promotes mitophagy in the RPE by activating the p62/Nrf2 pathway, while in DR, ROS may suppress mitophagy by inhibiting the FOXO3-pink1/parkin signaling pathway. Increasing evidence shows that oxidative stress injury, mitophagy, and damage to the RPE play an important role in the progression of retinal degenerative diseases. However, currently, the therapeutic strategy for AMD and DR predominantly focusing on anti-neovascularization and reducing permeabilization of abnormal vascular with anti-vascular endothelial growth factor (VEGF) drugs, and though some anti-inflammation drugs are also in the clinical trials for treating AMD, the results are unsatisfactory. The directly or indirectly regulating mitophagy may assist in maintaining mitochondrial homeostasis and promote the survival of the RPE. Therefore, investigating the key molecular targets of mitophagy and effectively regulating mitophagy may provide a novel therapeutic strategy for the retinal degenerative diseases.

Increasing evidence shows that mitophagy might play an important role in the pathogenesis of retinal degeneration, yet how to efficiently regulate mitophagy has become an inevitable question. Firstly, there are multiple signal pathways involving in mitophagy, which may interact with each other, and different stress stimuli may trigger various pathways in mitophagy. Therefore, it is necessary to decipher the exact molecular mechanism of mitophagy under the different stress damages. Second, the interaction between mitophagy-related proteins and receptors on the outer membrane of mitochondria regulating mitophagy is still not fully clarified. Currently, there is no highly efficient drug specifically regulating the process of mitophagy, but long-term utilization of broad-spectrum compounds may produce severe side effects on mitochondrial biology and cell function. In addition, mitophagy-induced quality control of mitochondria should rely on the dynamics of mitochondria and the function of mitochondrial network, but the coordination between mitophagy and other quality control pathways of mitochondria still need to be further investigated. Therefore, to decipher the signaling pathway and verify the key molecular factors regulating mitophagy in RPE cells, especially targeting ROS-related mitophagy, will be future research directions for looking for the promising treatment for retinal degenerative diseases.

## Data Availability

Not applicable.
